# Genome-wide DNA methylation changes upon DOT1L inhibition in hormone-responsive breast cancer cells

**DOI:** 10.3389/fcell.2023.1308025

**Published:** 2023-11-30

**Authors:** Giorgio Giurato, Ilaria Terenzi, Francesco Chiuso, Annamaria Salvati, Francesca Rizzo, Roberta Tarallo, Alessandro Weisz, Giovanni Nassa

**Affiliations:** ^1^ Laboratory of Molecular Medicine and Genomics, Department of Medicine, Surgery and Dentistry “Scuola Medica Salernitana”, University of Salerno, Baronissi, Italy; ^2^ Genome Research Center for Health - CRGS, Baronissi, Italy; ^3^ Department of Molecular Medicine and Medical Biotechnologies, University Federico II, Naples, Italy; ^4^ Molecular Pathology and Medical Genomics Program, Division of Oncology, AOU 'S. Giovanni di Dio e Ruggi d'Aragona', Università di Salerno, Salerno, Italy

**Keywords:** breast cancer, estrogen receptor alpha, DOT1l, epigenetics, methylation, reduced representation bisulfite sequencing

## Introduction

Breast cancer (BC) is a heterogeneous disease characterized by several molecular subtypes that differ in clinical behaviors and response to current therapies (Zardavas et al., 2015). Approximately 75% of BCs are estrogen receptor alpha positive (ERα+) and responsive to ovarian hormones (Chen et al., 2008). Hormone-activated ERα binds regulatory sites on chromatin (Cicatiello et al., 2010), where it assembles in large functional multiprotein complexes and exerts a direct control on target gene transcription, thus promoting breast carcinogenesis.

Given the strong dependency of a large fraction of BCs to the estrogen–ERα axis, endocrine therapy (ET) has been developed and implemented to suppress hormonal signaling by blocking ERα activity. ET makes use of selective ERα modulators (selective estrogen receptor modulators: SERMs, such as tamoxifen) or degraders (selective estrogen receptor degraders or downregulators: SERDs, such as ICI/fulvestrant) and aromatase inhibitors (AIs) (Patel and Bihani, 2018). Despite ET efficacy, one-third of patients develop *de novo* or acquired resistance, resulting in relapse and metastatic disease. Overall, only a minority (∼10%) of ET-resistant BCs show loss of ERα expression, while a sizable fraction retains the receptor. In the latter cases, resistance to therapy can occur in different ways: genetic changes, generating constitutive ERα signaling, altered receptor interactions with transcriptional coactivators/corepressors, or engagement of compensatory crosstalk with other oncogenic signaling pathways (Hanker et al., 2020).

Considering receptor interactions, ERα molecular partners are endowed with different functions (Nassa et al., 2019a) and comprise co-regulators (Ambrosino et al., 2010; Tarallo et al., 2011; Cirillo et al., 2013) and epigenetic modulators (Nassa et al., 2019b) that drive gene expression changes underlying BC development and progression. The histone H3 lysine 79 methyltransferase disruptor of telomeric silencing 1-like (DOT1L) is a recently identified ERα interactor (Nassa et al., 2019b) of particular interest, as it modulates mono-, di-, and tri-methylation of lysine 79 of histone H3 (H3K79), a key epigenetic modification controlling chromatin remodeling. It is also involved in deregulation of gene transcription in several cancer types (Salvati et al., 2019; Alexandrova et al., 2022a), and the therapeutic potential of DOT1L inhibition in BC has been demonstrated, together with its functional interplay with other essential BC genes, such as MEN1, revealing a new vulnerability of therapy-sensitive and -resistant breast and ovarian cancers (Alexandrova et al., 2022b; Salvati et al., 2022).

Together with histone modifications, DNA methylation represents a key epigenetic mechanism for regulation of gene expression in both normal and cancerous cells, including the mammary epithelium, holding great promise for expanding the range of therapeutic opportunities for personalized medicine. Usually, gene promoter CpG islands acquire abnormal hypermethylation or hypomethylation, resulting in transcriptional silencing of or activation of genes, respectively. However, methylation changes often do not reside on CpG islands but are spread away into shores, shelves (2–4 Kb from island), and open sea regions. Thus, DNA methylation is a potential candidate as a diagnostic and/or prognostic marker in BC, endowing conceivable implications for the clinical management of patients affected by this disease (Stefansson and Esteller, 2013).

Moreover, the molecular mechanisms that control DNA methylation in hormone-responsive BC, together with the impact of these epigenetic changes on the clinical outcome and response to pharmacological regimens, in particular ET, are still unclear and need further characterization (Garcia-Martinez et al., 2021). Interestingly, a strong correlation between DNA and histone methylation has been proposed and debated, implying a close functional relationship between these two epigenetic marks (Li et al., 2021). It has become apparent that DNA methylation and histone modification pathways can be dependent on one another and that this crosstalk can be mediated by biochemical interaction. Indeed, the molecular crosstalk between DNA and histone methylation on the cellular epigenome has potential implications in cancer development, progression, and response/resistance to therapies (Li et al., 2021).

Therefore, since the estrogen receptor is the master regulator of estrogen signaling in hormone-responsive BCs, where its expression is also regulated by epigenetic mechanisms, including DNA methylation, and that DOT1L associates with ERα in BC cell chromatin as a component of a multiprotein regulatory complex, we investigated the functional impact of ERα and DOT1L pharmacological inhibition on global DNA methylation in the same hormone-responsive BC cell model, generating a dataset of differentially methylated CpGs and genomic regions useful in understanding the contribution of this epigenetic pathway on the regulation of transcriptional pathways associated with BC progression.

## Methods

### Cell culture and DNA extraction

The human luminal ERα-positive MCF-7 breast cancer cell line (ATCC HTB-22) was purchased from the American Type Culture Collection (ATCC). MCF-7 was cultured in Dulbecco’s modified Eagle medium (Sigma-Aldrich) supplemented with 10% FBS (HyClone, Milan, Italy) and 100 U/mL penicillin, 100 mg/mL streptomycin, and 250 ng/mL amphotericin-B. MCF-7 cells were routinely tested for *Mycoplasma* contamination by using a PCR *mycoplasma* detection kit (ABM, Richmond, BC, Canada). Genomic DNA was extracted from three independent biological replicates of MCF-7 cells in exponentially growing condition, treated with 6.4 μM of a selective DOT1L inhibitor, the EPZ004777, from here on EPZ (S7353, Selleckchem), for 6 days or with 100 nM of the selective estrogen receptor degrader, fulvestrant (ICI182, 780), namely, ICI, for 3 days. To this aim, cell pellets were resuspended in two volumes of lysis buffer (10 mM Tris EDTA pH 8.4, 100 mM NaCl) supplemented with 3% sodium dodecyl sulfate and proteinase K (Thermo Fischer Scientific) and incubated for 15 min at 60°C under gentle shaking. Then, saturated NaCl was added, and the samples were centrifuged for 15 min at 25,000 rpm at 4 °C. The supernatants were then collected, diluted in two volumes of ethanol, and centrifuged for 10 min at 25,000 rpm at 4 °C. The DNA pellets were washed, dissolved in nuclease-free water, and quantified. Three independent biological replicates of MCF-7 cells following 6 or 3 days of treatment with appropriate vehicle (DMSO) were used as control and treated in parallel. DNA purity was determined by using the NanoDrop spectrophotometer ND-2000 (Thermo Fischer Scientific) through the evaluation of the absorbance ratio A260/A280. DNA concentration was determined by using the Quant-iT dsDNA High-Sensitivity Assay Kit and a Qubit Fluorometer (Life Technologies) and its quality and integrity assessed using the Agilent 4200 TapeStation System (Agilent Technologies).

### Reduced-representation bisulfite sequencing (RRBS) library preparation and sequencing

RRBS libraries were constructed according to the manufacturer’s instructions. In detail, 2 μg of genomic DNA was used for each library preparation. DNA was digested with the MspI enzyme (CCGG site) at 37°C for 16 h for each sample. The digested products were purified using the GeneJET PCR Purification Kit (Thermo Fisher Scientific), and libraries were prepared using the TruSeq Library Prep Kit (Illumina). The recovered DNA was treated with the EZ DNA Methylation-Gold Kit (Zymo Research) for bisulfite conversion. The converted DNA was amplified using PfuTurboCxHotstart DNA Polymerase (Agilent Technologies). Quantification of amplified fragments was assessed by the Agilent 4200 TapeStation (Agilent Technologies). Each DNA library was analyzed by paired-end sequencing of reads (2 × 75 cycles) on the Illumina NextSeq 500 sequencing system.

### Bioinformatics and functional annotation analyses

Quality assessment of the reads was performed using the FastQC quality control tool version 0.11.9 (https://www.bioinformatics.babraham.ac.uk/projects/fastqc/). Trim Galore software version 0.6.5 was used to perform adapter- and quality trimming, setting RRBS paired mode, in order to decrease methylation call errors arising from poor-quality data (https://www.bioinformatics.babraham.ac.uk/projects/trim_galore/). Alignment against the reference genome (GRCh38/hg38) was performed using Bismark version 0.22.3 (Krueger and Andrews, 2011) and Bowtie2 (Langmead and Salzberg, 2012) using default parameters. Methylation calls, sample correlation, and descriptive statistics on samples and differentially methylated CpG sites were performed using MethylKit version 1.17.5 (Akalin et al., 2012) using default parameters. CpGs were considered differentially methylated with a q-value≤ 0.05 and delta beta (|Db|) ≥ 0.10. Genomic regions with differentially methylated CpG sites were identified using DMRFinder (Gaspar and Hart, 2017) with default parameters. Differentially methylated regions were defined with a |Db|≥0.10 and an FDR≤ 0.05. Annotation of differentially methylated CpGs and differentially methylated regions on the genome was performed using the script annotatePeaks.pl of Homer software version 4.11 (Heinz et al., 2010), while annotation of CpGs on CpG islands, N- or S-shore, and N- or S-shelves was performed intersecting the CpGs identified in each sample, against the CpG island track downloaded from the Genome Browser. A circos plot was generated using ClicO FS (Cheong et al., 2015). Integration of differentially methylated CpGs with Nascent-Seq data, described by Nassa et al*.* (E-MTAB- 6,871) (Nassa et al., 2019b), was performed using R (version 4.2.2) scripts, considering the combination of upregulated transcripts—hypomethylated promoter region and downregulated transcripts—hypermethylated promoter region.

### Functional annotation analysis

A functional annotation analysis was performed with ShinyGO version 0.76.3 (Ge et al., 2020) setting the following parameters:

• Species: Human
• FDR cutoff: 0.05
• Pathway database: hallmark. MSigDB

Only hallmark categories showing a statistically significance according to the parameters used were reported.

### Code availability

The following software and versions were used for quality control and data analysis:

1) For methylation calls, sample correlation, and descriptive statistics on samples and differentially methylated CpGs were performed, MethylKit version 1.17.5 was used: http://bioconductor.org/packages/release/bioc/html/methylKit.html
2) For identification of genomic regions with differentially methylated CpG sites, DMRFinder was used: https://github.com/jsh58/DMRfinder
3) For the annotation of differentially methylated CpGs and differentially methylated regions on the genome, Homer software version 4.11 was used: http://homer.ucsd.edu/homer/
4) Functional analysis was performed by using ShinyGO version 0.76.3: http://bioinformatics.sdstate.edu/go/
5) Integration of data and statistical analyses were performed using R4.2.2:www.r-project.org
6) The circos plot was generated using ClicO FS: http://clicofs.codoncloud.com

## Data analysis

**FIGURE 1:**
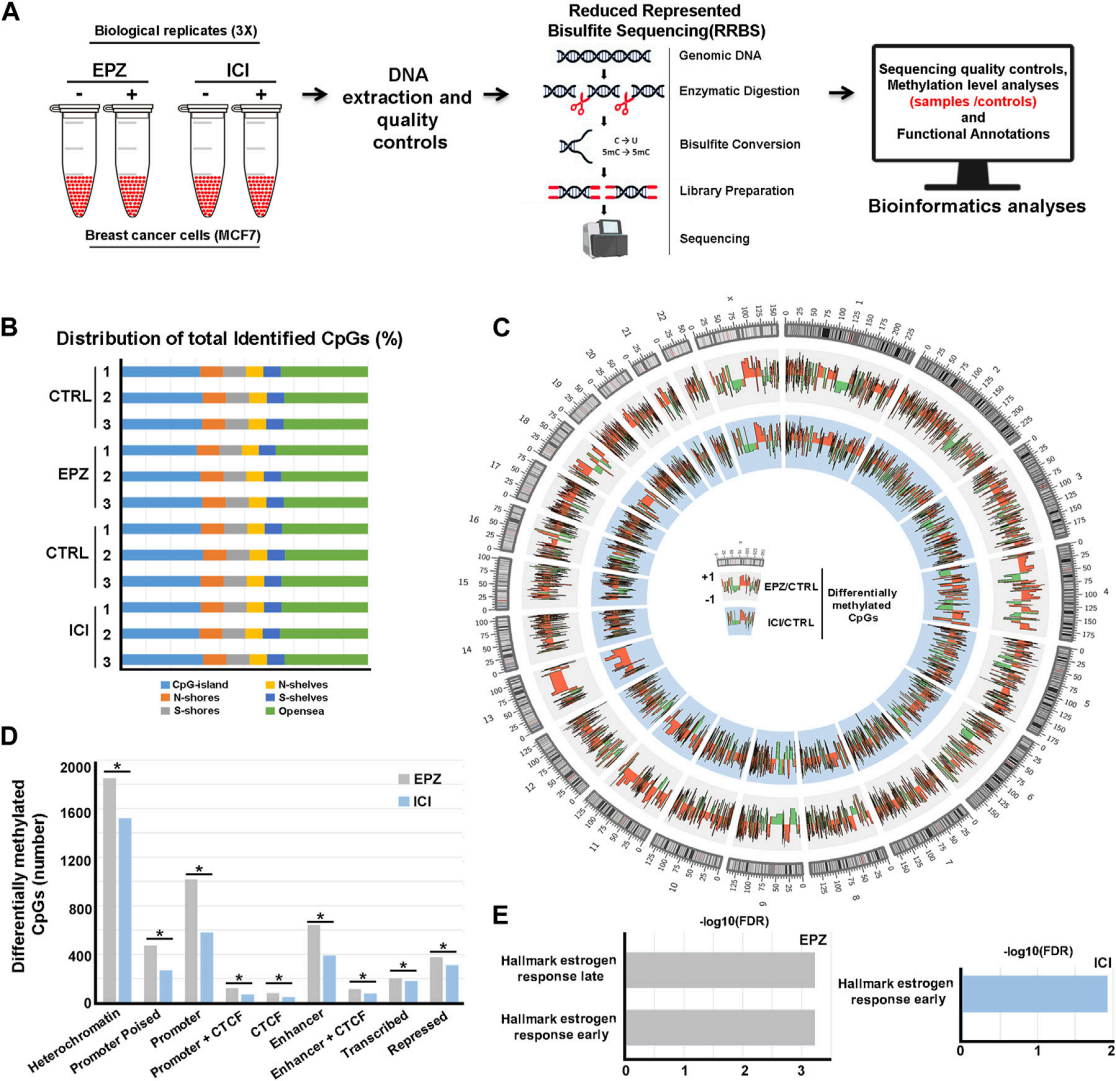
Analysis of DNA methylation profiles following EPZ and ICI treatments. **(A)** Summary of the experimental workflow applied to generate DNA methylation profile datasets. **(B)** Bar chart showing CpG distribution (shore: 0–2 Kb, shelves: 2–4 Kb, and open sea:>4 Kb upstream or downstream: S from the CpG island) in the three distinct biological replicates of MCF-7 cells treated with EPZ, ICI, or vehicle (CTRL). **(C)** Circos plot depicting differentially methylated CpGs obtained by RRBS between treated (EPZ: gray or ICI: light blue) and control samples in MCF-7 cells. **(D)** Bar chart showing the distribution of differentially methylated CpGs (EPZ: gray and ICI: light blue) on the genome. * (*p* < 0.01) denotes a statistically significant association between the differentially methylated CpGs and chromatin segmentation in the same BC cell models. **(E)** Bar chart showing statistically significant hallmarks, according to the gene set enrichment analysis (GSEA), involving differentially regulated transcripts from Nassa et al. (2019b) and harboring differentially methylated CPGs in the promoter region upon treatment with EPZ (gray) and ICI (light blue).

Based on the aforementioned observations, we mapped the DNA methylation changes following ERα and DOT1L inhibition. To this aim, BC cells were treated with either EPZ (Daigle et al., 2011), a selective DOT1L inhibitor, or ICI (Garcia-Martinez et al., 2021), a pure antiestrogen commonly used for ET, and subjected to Reduced-Representation Bisulfite sequencing (RRBS) and subsequent bioinformatics and functional annotation analyses (Figure 1A). In detail, three independent biological replicates of exponentially growing ERα+ MCF-7 cells were treated with vehicle only (controls), with 6.4 μM EPZ for 6 days or with 100 nM ICI for 3 days. These time-points were selected based upon the kinetics of each drug's response in these cells determined previously in these same cells (Nassa et al., 2019b). DNA was then extracted and purified before RRBS library preparation and sequencing. Bioinformatics analyses resulted in the initial identification of ∼5 million methylated CpGs/sample on average with minimum 10X read depth, according to the analytical steps detailed in the Methods section. Considering their distribution, most of them are located within CpG islands or harbored by N- or S-shore or -shelf regions and the remaining in non-annotated genomic locations (“open sea”, Figure 1B). Differential methylation analysis between treated and untreated samples was then employed to identify hypo- and hyper-methylated CpGs. In this way, we observed 5,690 hyper- and 2,280 hypo-methylated CpGs following treatment with EPZ and 3,814 hyper- and 1963 hypo-methylated CpGs following treatment with ICI (Figure 1C and Supplementary Material S1, Supplementary Table S1 and Supplementary Material S2, Supplementary Table S2). Since drug-responsive DNA methylation sites are most frequently clustered into short regions, we applied a bioinformatics approach to determine differentially methylated regions (DMRs). This analysis revealed 268 hyper- and 36 hypo-methylated and 136 hyper- and 38 hypo-methylated regions in response to EPZ and ICI, respectively (Supplementary Material S3, Supplementary Table S3 and Supplementary Material S4, Supplementary Table S4, respectively). When considering differentially methylated regions, an overlap of about 5% between the two drug treatments was observable, with the rest being specific to each compound. As shown in Figure 1D, functional annotation of the two differentially methylated CpG datasets, performed considering the chromatin segmentation in the same BC cell models (Taberlay et al., 2014), revealed their statistically significant (*p* < 0.01) prevalent location in genetic regulatory elements, such as gene promoters and enhancers and heterochromatic regions. The significance of the association between the differentially methylated CpGs and the different chromatin states was verified using the poverlap tool (https://github.com/brentp/poverlap) by performing 1.000 permutations. Considering the former result, we integrated the information gained from differential methylation analyses with gene expression data previously obtained by Nascent-Seq (Nassa et al., 2019b) to obtain more insight into the biological significance of these findings. By merging promoter methylation and transcriptomics data, we observed 28 and 75 transcripts regulated by EPZ and ICI, respectively, in combination with significant changes in DNA methylation patterns. Functional analysis, conducted using the gene set enrichment analysis (GSEA) tool, revealed their involvement in early and late estrogen response gene pathways (Figure 1E), confirming and integrating our previous results (Nassa et al., 2019b; Salvati et al., 2019). The epigenetic datasets reported here are now useful to investigate, in greater detail, the biological significance and molecular mechanisms underlying the epigenetic actions, in particular changes in DNA methylation, of DOT1L and ERα in BC cells, in view of the significant effects of pharmacological inhibition of the two factors on the proliferation and survival of these cells.

## Technical validation

**FIGURE 2:**
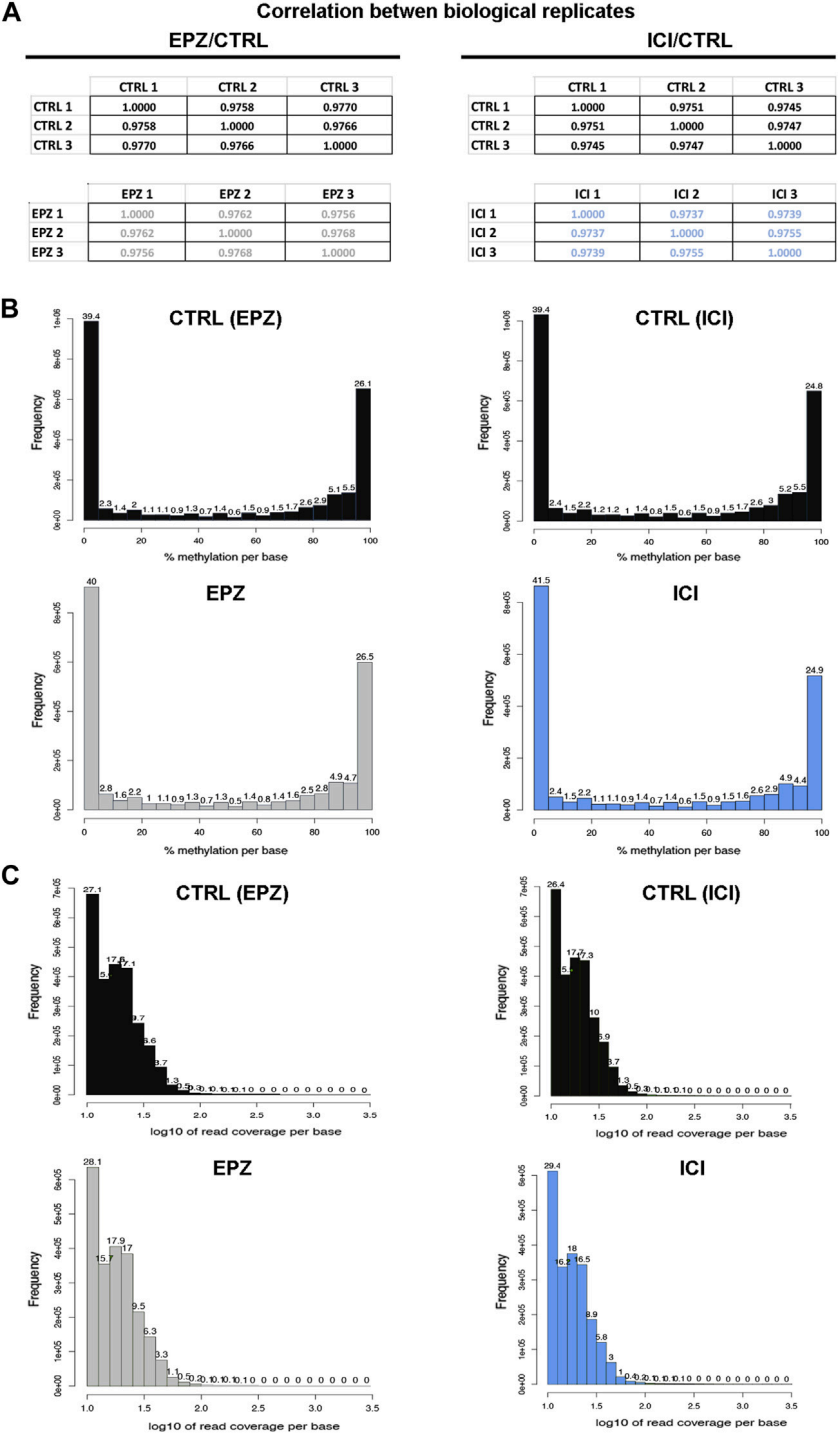
Quality controls of the experimental procedure. **(A)** Matrices showing Pearson’s correlation computed among the three biological replicates considered for bioinformatics analysis. **(B)** Representative bar chart showing the percentage of methylation distribution for the biological replicates of control (CTRL: black) and treated samples (EPZ: gray and ICI: light blue). **(C)** Representative bar chart showing read coverage per base information for the biological replicates of control (CTRL: black) and treated samples (EPZ: gray and ICI: light blue).

To ensure the quality and robustness of the data presented here, our datasets were generated from three independent biological replicates for each experimental condition analyzed, by using cell cultures processed independently from authenticated and mycoplasma-free exponentially growing MCF7 cells. This cell line represents the most widely used model of luminal A BC, and pivotal works using this cell line continue unabated. MCF-7 cell lines have dramatically supported the course of BC research and have contributed to clinically relevant discoveries able to improve the outcome of patients affected by this disease (Lee et al., 2015), indicating that the results shown here are unlikely to be considered merely cell line-specific, as already reported (Tarallo et al., 2011). Moreover, each RRBS biological replicate was performed independently, and both controls and samples were analyzed in parallel. Quality check of the biological replicates was performed by computing the Pearson correlation coefficient (r) among the analyzed samples, considering the number of detected CpGs for each sample and the corresponding beta value (Figure 2A). This analysis highlighted a strong correlation among the replicates (Pearson’s r ≥ 0.9737), giving strength to the biological findings. Concerning methylation percentages, typically a histogram representing it should have two peaks on both ends since in any given cell, any given base is either methylated or not. Therefore, looking at many cells should yield a similar pattern, where it is possible to see lots of locations with high methylation and lots of locations with low methylation (Akalin et al., 2012). This also occurs in our datasets; in fact, the analysis of average DNA methylation on detected CpGs showed a bimodal distribution, with peaks at 0%–10% methylation and 95%–100% methylation (Figure 2B) as expected. Then, to ensure that each sample was not affected by PCR duplication, we plotted the read coverage per base information (Figure 2C), which showed that this problem was avoided in the generation of our datasets. Moreover, from a biological point of view, we also confirmed and expanded our previous results concerning DOT1L pharmacological blockade of ET-sensitive and -resistant BC cell proliferation, revealing, also here, an impact comparable to that of ICI on the estrogen receptor signaling partway via deregulation of early and late estrogen response genes (Figure 1E). Among them are worth mentioning RARA, which results in competitive binding activity at nearby or overlapping cis-regulatory elements with ERα (Hua et al., 2009) and the enhanced expression of which induces epithelial-to-mesenchymal transition and disruption of mammary acinar structures guiding malignant transformation during mammary tumorigenesis (Doi et al., 2015); CALCAR that belongs to a gene signature regulated by the estrogen, the expression of which changes in ET resistance phenomena (Cheng et al., 2020); and TAM1, as its expression induces changes in invasion, migration, epithelial–mesenchymal transition, and cancer stem cell characteristics in BC cells (Xu et al., 2016).

## Conclusion

Considering the results obtained, we conclude that the data reported in this data descriptor represent high-quality next-generation sequencing methylation data that are biologically valid and should be useful for future reuse in studies that seek to understand the impact of histone and DNA methylation in BC. It will also be an important resource for future comparative studies of BC DNA methylation patterns involved in response and resistance to ET where integration with epigenomics data could be needed.

## Data Availability

The datasets presented in this study can be found in online repositories. The raw sequencing data related to CTRLs, EPZ, and ICI methylation profiles have been deposited to Figshare archive (https://doi.org/10.6084/m9.figshare.22140335.v5) and in the EBI ArrayExpress database (http://www.ebi.ac.uk/arrayexpress) with accession number E-MTAB-12635.
